# Duration of Symptom Relief Between Injections for AbobotulinumtoxinA (Dysport®) in Spastic Paresis and Cervical Dystonia: Comparison of Evidence From Clinical Studies

**DOI:** 10.3389/fneur.2020.576117

**Published:** 2020-09-25

**Authors:** Alberto Esquenazi, Mauricio R. Delgado, Robert A. Hauser, Philippe Picaut, Keith Foster, Andreas Lysandropoulos, Jean-Michel Gracies

**Affiliations:** ^1^Department of Physical Medicine and Rehabilitation, MossRehab Gait and Motion Analysis Laboratory, Elkins Park, PA, United States; ^2^Neurology and Neurotherapeutics Department, University of Texas Southwestern Medical Center and Scottish Rite Hospital for Children, Dallas, TX, United States; ^3^University of South Florida Parkinson's Disease and Movement Disorders Center of Excellence, Tampa, FL, United States; ^4^Ipsen Pharma, Cambridge, MA, United States; ^5^Ipsen Bioinnovation, Abingdon, United Kingdom; ^6^Ipsen, Boulogne-Billancourt, France; ^7^UR 7377, Université Paris-Est Créteil, Service de Rééducation Neurolocomotrice, Albert Chenevier-Henri Mondor Hospital, Créteil, France

**Keywords:** abobotulinumtoxinA, botulinum toxin-A, duration of response, spasticity, cervical dystonia, treatment

## Abstract

**Background:** Botulinum toxin-A is a well-established treatment for adult and pediatric spastic paresis and cervical dystonia. While guidelines and approved labels indicate that treatment should not occur more frequently than every 12 weeks, studies and real-world evidence show that the timing of symptom recurrence between treatments may vary.

**Methods:** We report retreatment criteria and response duration (retreatment intervals) from four pivotal, double-blind, placebo-controlled studies with open-label extensions involving patients treated with abobotulinumtoxinA (aboBoNTA) for upper limb (NCT01313299) or lower limb (NCT01249404) spastic paresis in adults, lower limb spastic paresis in children (NCT01249417), and cervical dystonia in adults (NCT00257660). We review results in light of recently available preclinical data.

**Results:** In spastic paresis, 24.0–36.9% of upper limb patients treated with aboBoNTA and 20.1–32.0% of lower limb patients did not require retreatment before 16 weeks. Moreover, 72.8–93.8% of aboBoNTA-treated pediatric patients with lower limb spastic paresis did not require retreatment before 16 weeks (17.7–54.0% did not require retreatment before 28 weeks). In aboBoNTA-treated patients with cervical dystonia, 72.6–81.5% did not require retreatment before 16 weeks.

**Conclusion:** AboBoNTA, when dosed as recommended, offers symptom relief beyond 12 weeks to many patients with spastic paresis and cervical dystonia. From recently available preclinical research, the amount of active neurotoxin administered with aboBoNTA might be a factor in explaining this long duration of response.

## Introduction

Botulinum neurotoxins (BoNTs) are potent toxins that inhibit neurotransmitter release, which results in flaccid paralysis underpinning their therapeutic use (1). Injection of BoNT into specific muscles of patients with movement disorders, such as dystonia and spastic paresis, causes muscle relaxation, which may lead to symptom relief and facilitate rehabilitation (2, 3).

While there are eight naturally occurring serotypes of BoNT in nature (4, 5), BoNT serotype A (BoNTA) has been the most commonly used in the clinic. There are currently three BoNTA products available worldwide: abobotulinumtoxinA (aboBoNTA; Dysport®, Ipsen, Paris, France), onabotulinumtoxinA (Botox®, Allergan, Irvine, USA), and incobotulinumtoxinA (Xeomin®, Merz Pharmaceuticals GmbH, Frankfurt, Germany). For each of these BoNTA products, there is a unique manufacturing process that results in each containing different excipients (6). As a result of these differences in formulation, each BoNTA has its own dosing guidelines and units of activity; however, it is important to note that the units of each product are not interchangeable ((7, 8), Dysport SmPC).

The effects of chemodenervation with all BoNTA products are transient, and repeat injections are usually necessary to maintain clinical benefit. In animal models, the duration of the BoNT-induced effect is determined by the amount of BoNTA delivered (9, 10). The minimum interval between BoNTA injections recommended by many regulatory authorities has historically been 12 weeks, although it has been suggested that this minimum interval was empirically proposed for putative immunological reasons based on retrospective clinical data and not on scientific grounds (11).

In that context, many patients experience loss of symptom relief before their next injection, which negatively impacts on both patients' and caregivers' quality of life. A recent survey of patients treated with a range of BoNTA products suggested that symptom recurrence between injections may affect as many as 83% of patients (12), with mean time to symptom recurrence reported as being from 9.3 weeks (13) to 12.8 weeks, and 53% of patients were getting symptoms back prior to 3 months. Hence, there is an unmet need for long-lasting symptom relief spanning the entire period between injections.

To date, few studies have assessed the time to retreatment during repeated injections of BoNTA (14). Four such studies have demonstrated the efficacy of aboBoNTA (Dysport®) in improving clinical outcomes and function across a number of indications; these include adults with upper or lower limb spastic paresis following a stroke or traumatic brain injury (15–17), children with lower limb spastic paresis as a result of cerebral palsy (18, 19), and adults with cervical dystonia (20). These trials established the clinical efficacy of aboBoNTA; a separate dose-ranging analysis in the upper limb reported dose-dependent improvements in spasticity and in active movement with aboBoNTA (21).

Here we evaluate the duration of the effect of aboBoNTA injections over repeated treatment cycles among patients who successfully completed one of the four, double-blind, open-label extension trials identified (15, 16, 18–20). We also review this data in light of recently available preclinical data describing the quantity and the activity of neurotoxin in available BoNTA products (22).

## Materials and Methods

Full details of the study methodologies are included in the original publications (15–20) and have been summarized in the “Methodology” section (see Supplemental Information).

### Patient Population

**Figure 1:**
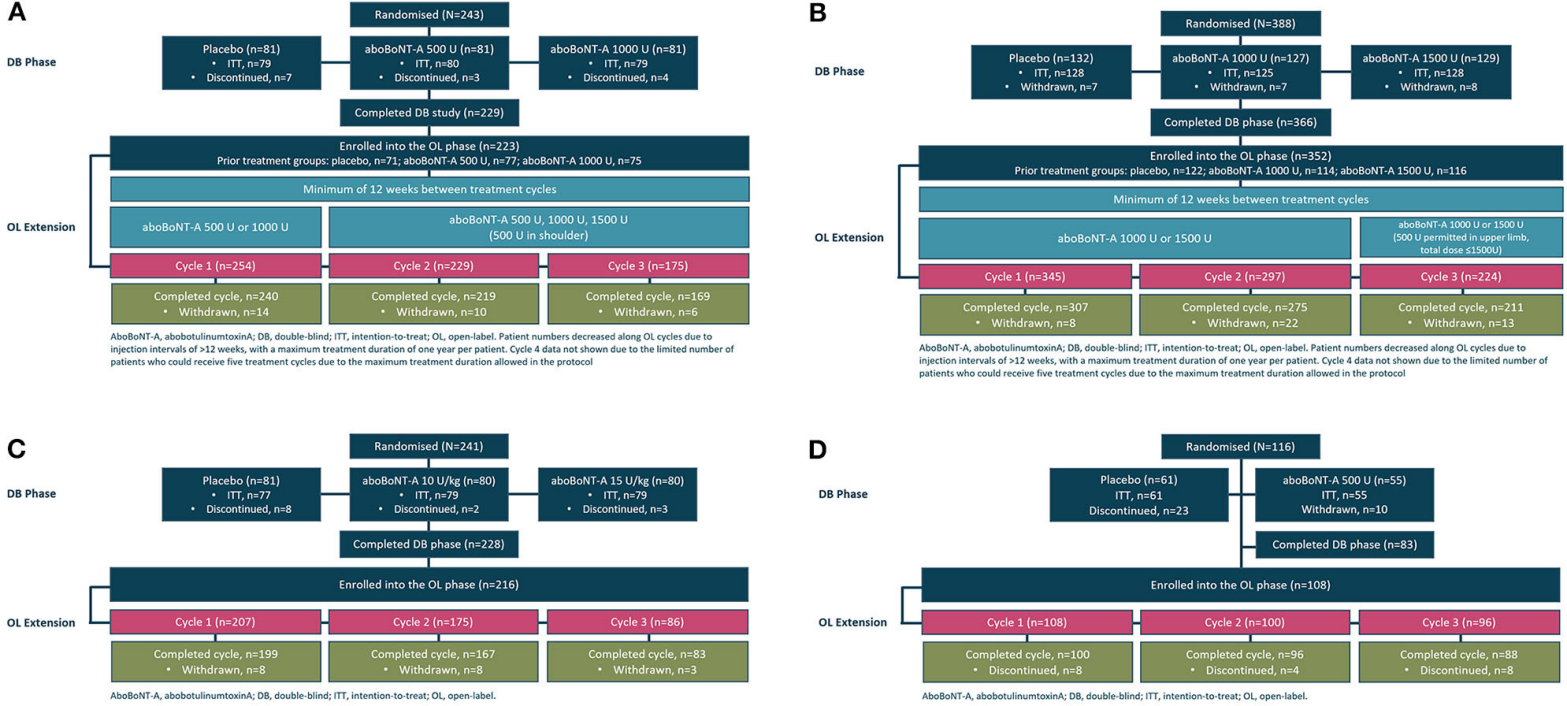
Study flow schematic illustrating patient flow from the double-blind to the open-label phases in the **(A)** adult upper limb spasticity study, **(B)** adult lower limb spasticity study, **(C)** pediatric lower limb spasticity study, and **(D)** cervical dystonia study.

The baseline demographics and the characteristics of the patients in each open-label extension phase of the four studies are reported in detail in the study publications (15–20) and are shown in Table 1, while data on patient disposition during the double-blind phase and the subsequent open-label extension of each study are displayed in Figures 1A–D. Adults with upper limb spasticity received 500, 1,000, or 1,500 U aboBoNTA, adults with lower limb spasticity received 1,000 or 1,500 U aboBoNTA, children with lower limb spasticity received a maximum total dose of 30 U/kg or 1,000 U aboBoNTA, whichever is the lower value, and adults with cervical dystonia received 500 U aboBoNTA (up to 1,000 U in the open-label phase). Criteria for retreatment differed between studies. For the adult upper limb and lower limb studies, retreatment was decided per the investigator's clinical judgment and was possible at weeks 12, 16, 20, and 24. In the upper limb study, if the patient had not demonstrated a decrease from baseline of at least one grade in the Modified Ashworth Scale (MAS) score in the primary targeted muscle group and had no improvement on the Physician's Global Assessment (PGA; i.e., a score ≤0) and if, based on the investigator's judgment, there was no unacceptable safety risk for the subject to receive the next treatment cycle, then the patient was injected on the same study day. An analogous algorithm was applied in the lower limb study, except that the MAS score was assessed in the gastrocnemius–soleus complex (GSC; knee extended). In both studies, if the patient had demonstrated improvement according to any of those criteria, it was left to the investigator's judgment whether the injection should happen on the same day or be postponed. In the pediatric lower limb study, if the patient had not demonstrated a decrease from baseline of ≥1 grade in the MAS score in the GSC at the ankle joint and had no improvement in PGA score (i.e., score ≤0), the patient was injected on the same study day (assuming that, in the investigator's judgment, there was no unacceptable safety risk). Finally, in the cervical dystonia study, the exact timing of retreatment was determined by the investigator based on clinical need. Further information on these studies can be found in the original publications and in the “Methodology” section (see Supplemental Information).

**Table 1:** Open-label phase baseline demographics and characteristics of patient population for each of the four studies.

**Characteristic**	**Study**			
	**AUL (*N* = 258)**	**ALL (*N* = 352)**	**PLL (*N* = 216)**	**CD (*N* = 131)**
Mean age, years (SD)	52.4 (13.9)	53.2 (12.7)	5.9 (3.3)	54.0 (12.3)
Sex, male, *n* (%)	166 (64.3)	239 (67.9)	130 (60.2)	50 (38.2)
Mean weight, kg (SD)	81.3 (18.6)	79.9 (16.5)	NR	74.0 (15.4)
Affected leg, left, *n* (%)	NA	194 (55.1)	NR	NA
Affected arm, left, *n* (%)	128 (49.6)	NA	NA	NA
Cause, *n* (%) Stroke TBI	230 (89.1) 28 (10.9)	309 (87.8) 43 (12.2)	NR	NA
Mean time since event, years (SD) Stroke TBI	5.1 (4.2) 9.9 (8.0)	4.5 (4.8) 9.2 (10.1)	NR NR	NA NA
Treatment naïve^a^, *n* (%)	116 (45.0)	226 (64.2)	NR	17 (13.0)

a *Treatment naive is defined as non-use of BoNTA <4 months prior to study entry*.

*ALL, adult lower limb; AUL, adult upper limb; CD, cervical dystonia; NA, not applicable; NR, not reported; PLL, pediatric lower limb; SD, standard deviation; TBI, traumatic brain injury*.

## Results

### Adult Upper Limb Spasticity

**Figure 2:**
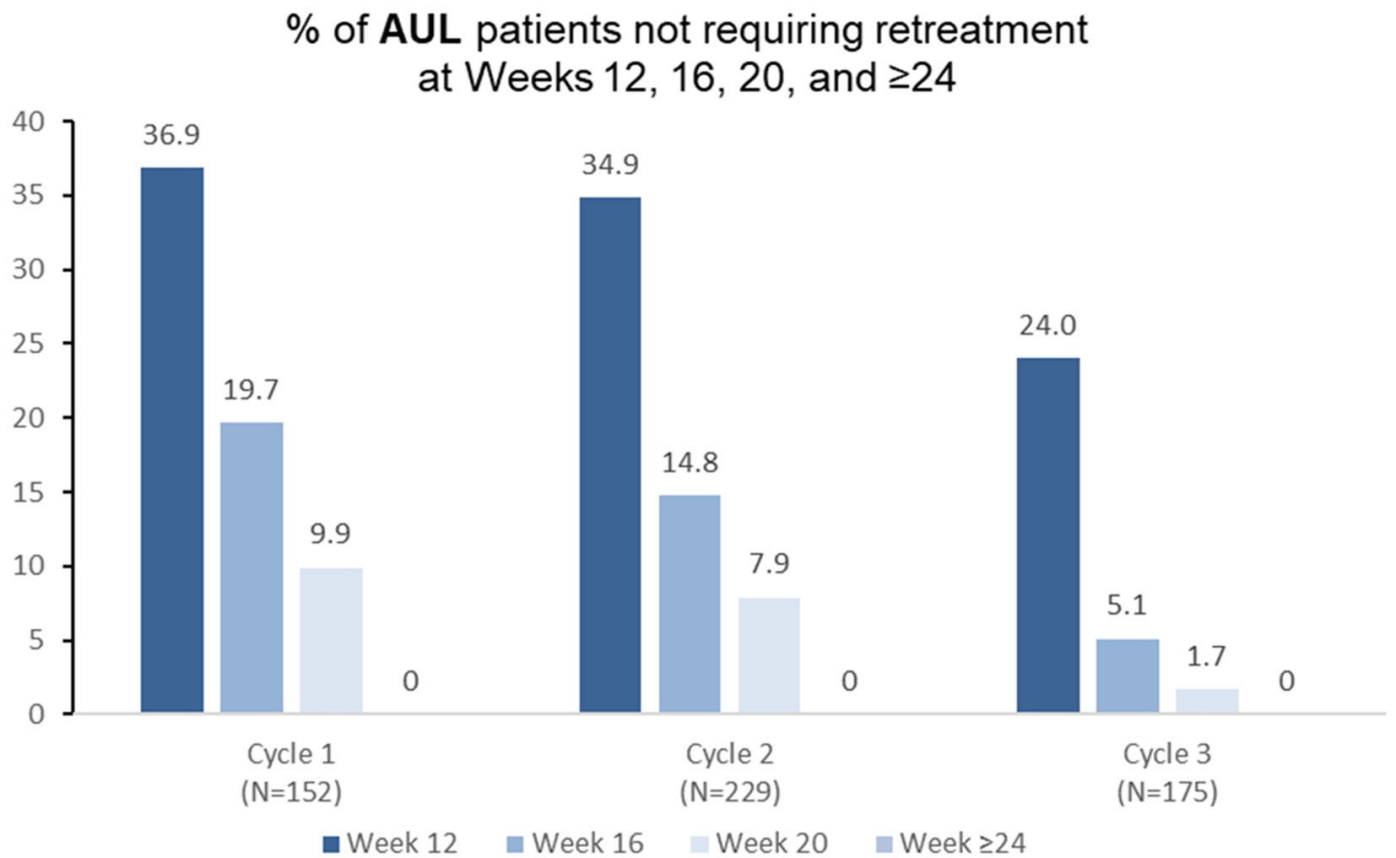
Proportion of patients with adult upper limb spasticity not requiring retreatment at different timepoints during each cycle in the open-label extension (OLE) phase [*n*.*b*. cycle 1 includes patients who were treated with aboBoNTA in the double-blind (DB) phase and entered the OLE. Cycles 2 and 3 include patients who received aboBoNTA in the OLE (including those who received placebo in the DB phase). Patients who were retreated at week 12 of each cycle are not shown].

Among patients who received aboBoNTA in the double-blind study and were treated at cycle 1 of the open-label extension (*n* = 152), over a third (36.9%) did not need retreatment before week 16, with 19.7% still not needing reinjection at week 16 and 9.9% not needing reinjection at week 20 (Figure 2). Similar patterns were observed in treatment cycle 2 of the open-label phase (*n* = 229), where 34.9% were re-injected at week 16 or later, and in treatment cycle 3 (*n* = 175), where 24.0% were re-injected at week 16 or later (Figure 2).

### Adult Lower Limb Spasticity

**Figure 3:**
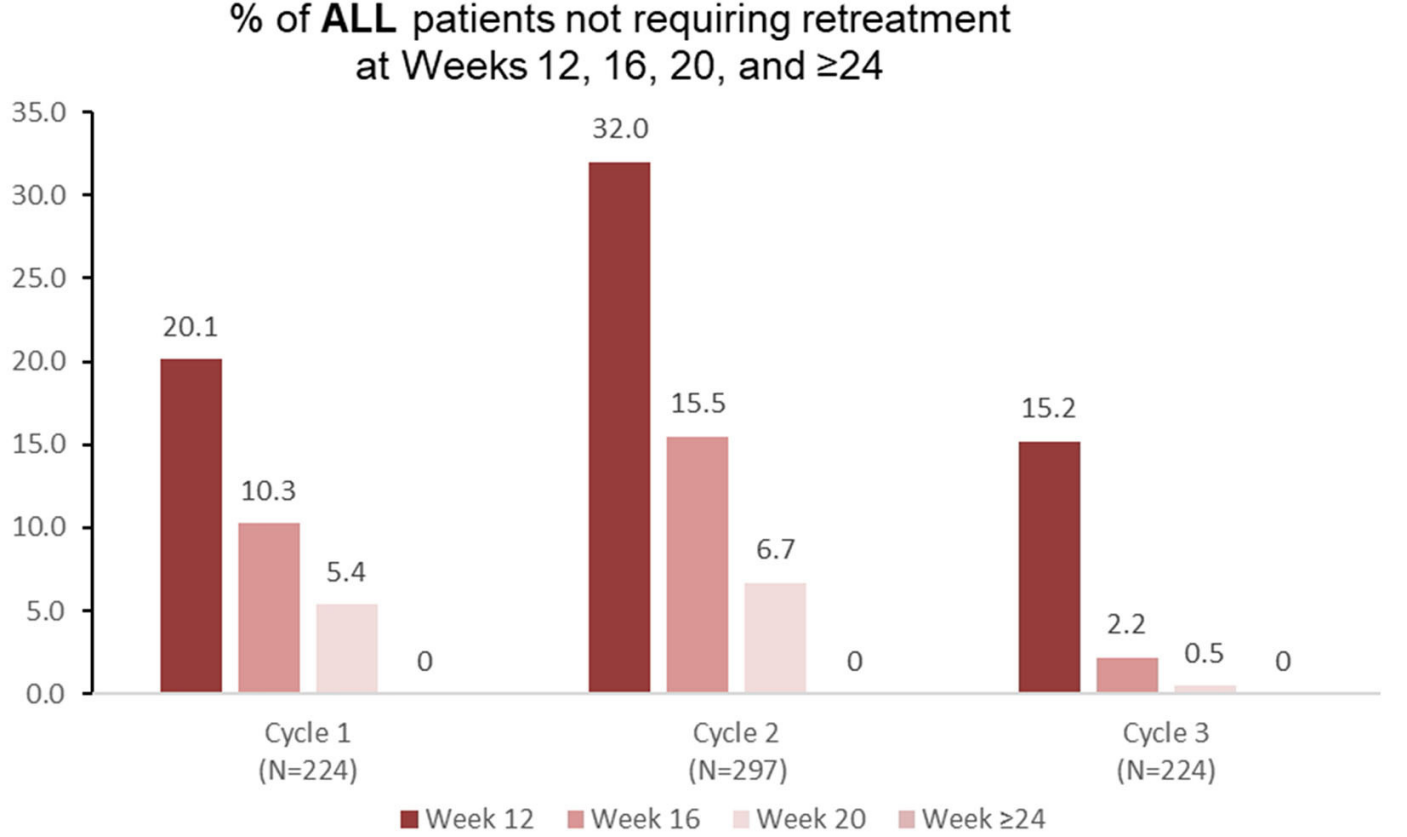
Proportion of patients with adult lower limb spasticity not requiring retreatment at different timepoints during each cycle in the open-label extension phase [OLE; *n*.*b*. cycle 1 includes patients who were treated with aboBoNTA in the double-blind (DB) phase and entered the OLE. Cycles 2 and 3 include patients who received aboBoNTA in the OLE (including those who received placebo in the DB phase). Patients who were retreated at week 12 of each cycle are not shown].

Among patients with lower limb spasticity who were reinjected at treatment cycle 1 of the open-label extension (*n* = 224), 20.1% did not require retreatment before week 16 (Figure 3), 10.3% did not require retreatment at week 16, and 5.4% did not require retreatment at week 20. Similar patterns were observed in treatment cycles 2 and 3 (Figure 3).

### Pediatric Lower Limb Spasticity

**Figure 4:**
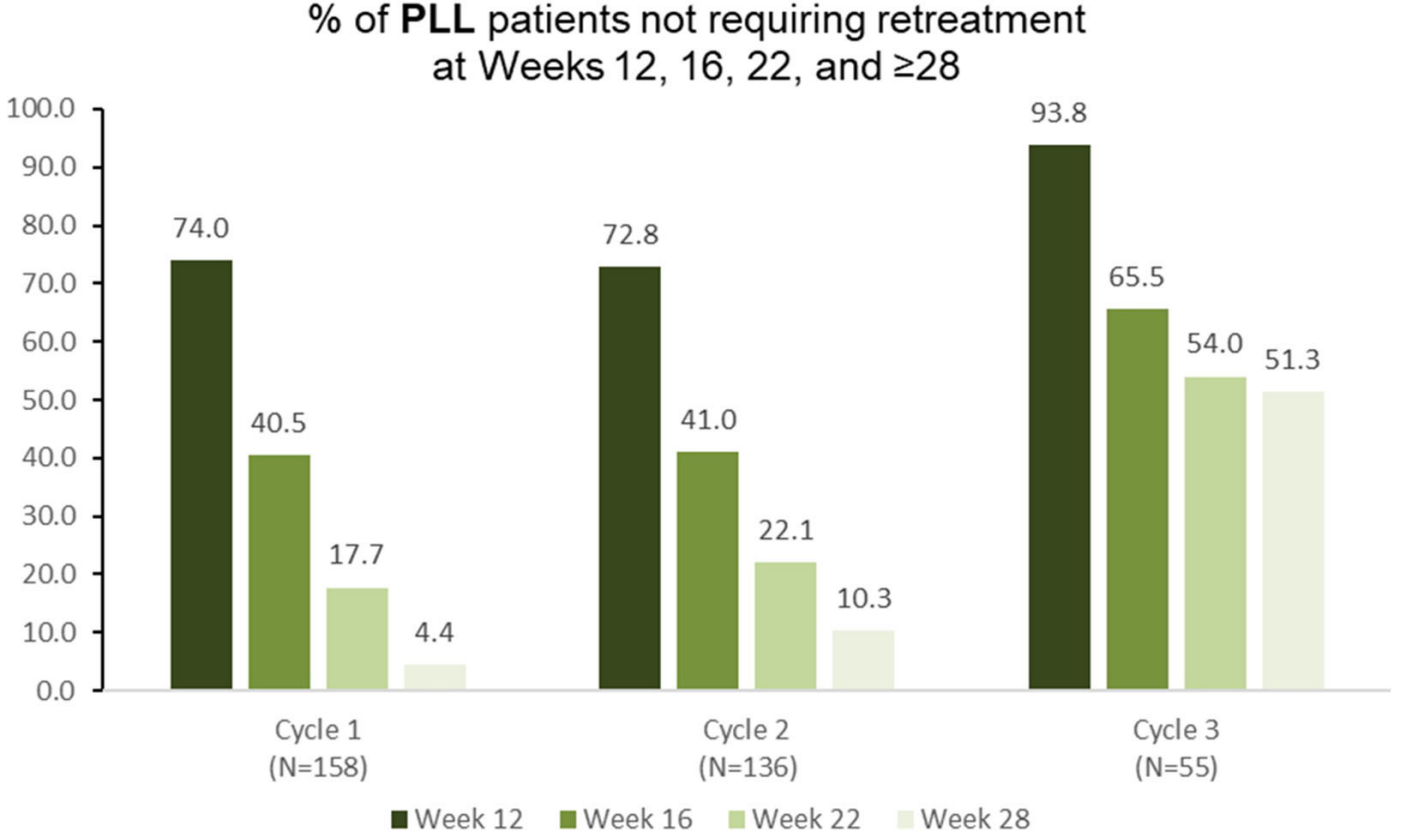
Proportion of patients with pediatric lower limb spasticity not requiring retreatment at different timepoints during each cycle in the open-label extension phase [OLE; *n*.*b*. cycle 1 includes patients who were treated with aboBoNTA in the double-blind (DB) phase and entered the OLE. Cycles 2 and 3 include patients who received aboBoNTA in the OLE (including those who received placebo in the DB phase). Patients who were retreated at week 12 of each cycle are not shown].

Among 158 pediatric patients with lower limb spasticity who received aboBoNTA (all doses) in the double-blind study, 74.0 and 40.5% did not require retreatment at weeks 12 and 16, respectively, with 17.7% of patients not requiring retreatment before week 28 (Figure 4). Similar results were observed in the second (*n* = 136) and in the third (*n* = 55) treatment cycles (Figure 4).

### Cervical Dystonia

**Figure 5:**
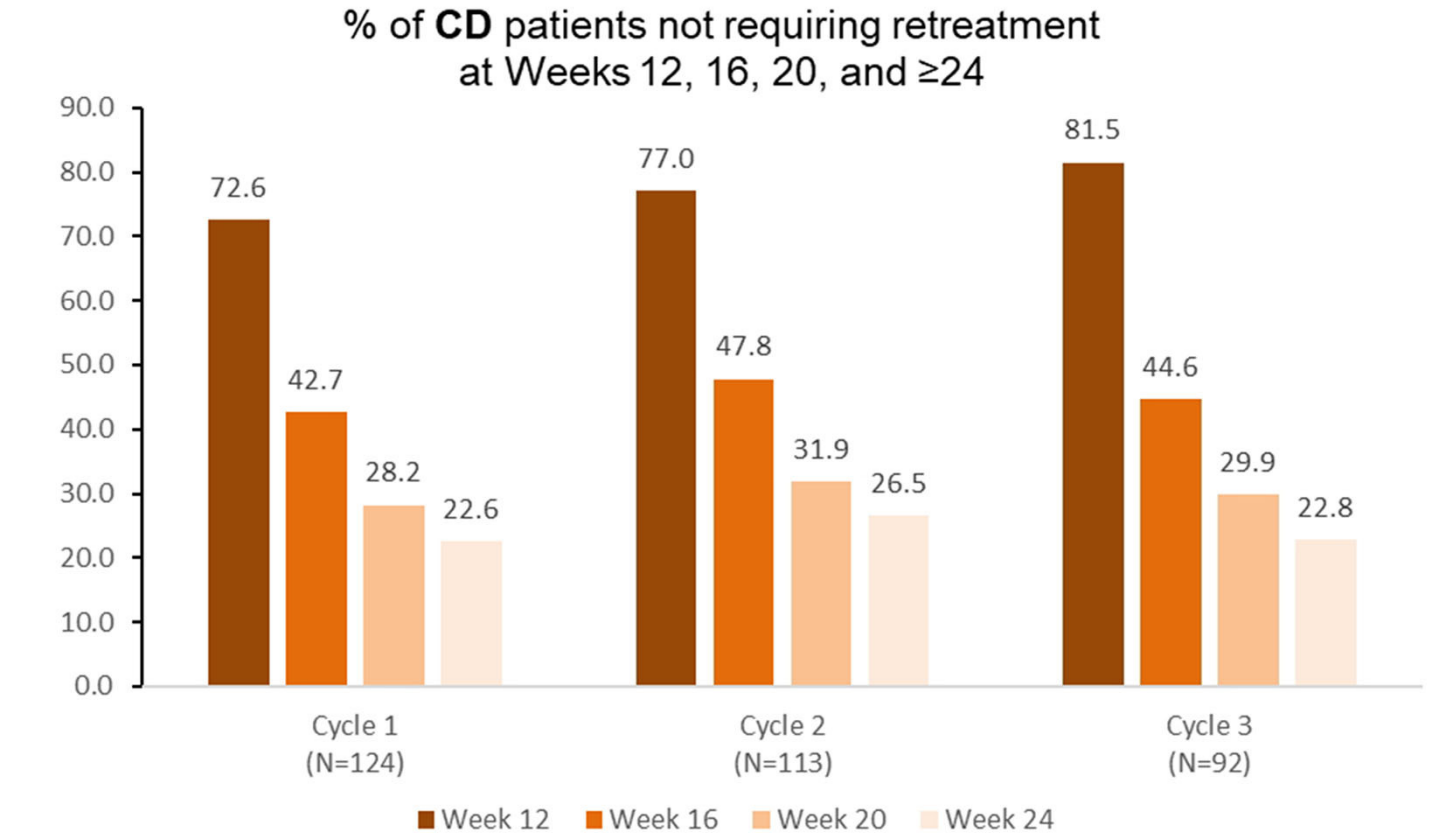
Proportion of patients with cervical dystonia not requiring retreatment at different timepoints during each cycle in the open-label extension phase [OLE; *n*.*b*. cycle 1 includes patients who were treated with aboBoNTA in the double-blind (DB) phase and entered the OLE. Cycles 2 and 3 include patients who received aboBoNTA in the OLE (including those who received placebo in the DB phase). Patients who were retreated at week 12 of each cycle are not shown. The cycle 3, week 20 value represents a mean of values taken at weeks 19 and 21].

Among patients with cervical dystonia receiving aboBoNTA and treated at treatment cycle 1 of the open-label extension (*n* = 124), 42.7% did not require retreatment before week 16 (Figure 5). Similar results were observed again during the second (*n* = 113) and the third (*n* = 92) retreatment cycles (Figure 5).

### Safety

In both adult upper and lower limb spasticity studies, the most frequently reported treatment-emergent adverse events (TEAEs) were muscular weakness, falls, and pain in the treated extremity (Table 2).

**Table 2:** Patients experiencing treatment-emergent adverse events (TEAEs; displayed by ≥2% of patients in any cycle) during the open-label phase of each of the four studies (safety populations).

**TEAEs**		**AUL**			**ALL**			**PLL**			**CD**	
	**Cycle 1,** ***n* (%)** **(*n* = 254)**	**Cycle 2,** ***n* (%)** **(*n* = 229)**	**Cycle 3,** ***n* (%)** **(*n* = 175)**	**Cycle 1,** ***n* (%)** **(*n* = 345)**	**Cycle 2,** ***n* (%)** **(*n* = 297)**	**Cycle 3,** ***n* (%)** **(*n* = 224)**	**Cycle 1,** ***n* (%)** **(*n* = 201)**	**Cycle 2,** ***n* (%)** **(*n* = 168)**	**Cycle 3,** ***n* (%)** **(*n* = 80)**	**Cycle 1,** ***n* (%)** **(*n* = 131)**	**Cycle 2,** ***n* (%)** **(*n* = 121)**	**Cycle 3,** ***n* (%)** **(*n* = 111)**
Any TEAE	102 (40.2)	62 (27.1)	47 (26.9)	140 (40.6)	97 (32.7)	47 (21.0)	82 (40.8)	46 (27.4)	14 (17.5)	107 (82)	84 (69)	67 (60)
Related TEAEs	18 (7.1)	8 (3.5)	5 (2.9)	43 (12.5)	23 (7.7)	7 (3.1)	16 (8.0)	4 (2.4)	2 (2.5)	68 (52)	68 (56)	67 (60)
Muscle weakness	9 (3.5)	2 (0.9)	2 (1.1)	22 (6.4)	12 (4.0)	6 (2.7)	NR	NR	NR	33 (25)	21 (17)	22 (20)
Fall	9 (3.5)	7 (3.1)	6 (3.4)	17 (4.8)	17 (5.7)	9 (4.0)	0	1 (0.6)	0	NR	NR	NR
Pain in extremity	6 (2.4)	6 (2.6)	2 (1.1)	11 (3.2)	4 (1.3)	2 (0.9)	1 (0.5)	1 (0.6)	0	13 (10)	9 (7)	5 (5)
Arthralgia	NR	NR	NR	6 (1.7)	6 (2.0)	1 (0.4)	NR	NR	NR	NR	NR	NR
Dysphagia	NR	NR	NR	1 (0.3)	6 (2.0)	1 (0.4)	NR	NR	NR	25 (19)	31 (26)	22 (20)
Asthenia	NR	NR	NR	9 (2.6)	6 (2.0)	4 (1.8)	NR	NR	NR	13 (10)	12 (10)	6 (5)
Bronchitis	0	1 (0.4)	4 (2.3)	NR	NR	NR	NR	NR	NR	NR	NR	NR
Dry mouth	NR	NR	NR	NR	NR	NR	NR	NR	NR	19 (15)	13 (11)	8 (7)
Headache	NR	NR	NR	NR	NR	NR	NR	NR	NR	13 (10)	10 (8)	8 (7)
Injection site pain	NR	NR	NR	NR	NR	NR	7 (3.5)	3 (1.8)	1 (1.3)	NR	NR	NR
Injection site papule	NR	NR	NR	NR	NR	NR	2 (1.0)	1 (0.6)	1 (1.3)	NR	NR	NR
Influenza-like illness	NR	NR	NR	NR	NR	NR	2 (1.0)	0	0	NR	NR	NR

*NR, not reported*.

In the pediatric lower limb spasticity study, the incidence of TEAEs was low; the only treatment-related TEAE reported by more than two patients was injection site pain (Table 2). There were no differences in the number of treatment-related TEAEs according to the dose administered and no trend toward a greater frequency of events with increasing number of cycles. In general, the proportion of patients reporting TEAEs tended to decrease with each treatment cycle in all spasticity studies.

In the cervical dystonia study, the incidence of TEAEs was considerably higher than that observed in the other studies, with the most frequently reported TEAEs being muscle weakness in the neck, dysphagia, neck/shoulder pain, dry mouth, headache, and asthenia (Table 2).

In all four pivotal clinical trials, the rate of neutralizing antibody formation or remote toxin spread associated with aboBoNTA treatment was low. In the adult upper limb (AUL) study, among those treated with aboBoNTA, neutralizing antibodies developed in 13 patients (two seroconverted in the double-blind phase and 11 seroconverted in the open-label extension). Three of these patients returned to negative by study end, and there was no suggestion of impact on abobotulinumtoxinA efficacy in patients with neutralizing antibodies, at baseline or throughout the study. No cases of neutralizing antibodies were detected in the adult lower limb (ALL) study, in either the double-blind or open-label phases, in spite of the fact that these studies used the highest dose of aboBoNTA (1,500 U) among any of the studies under consideration in this publication. In the pediatric lower limb (PLL) study, neutralizing antibodies were found in four aboBoNTA-treated patients (all in the open-label phase) without any associated efficacy or safety concerns. In the cervical dystonia (CD) study, only one patient developed neutralizing antibodies during the study's final treatment cycle. The number of adverse events suggestive of remote toxin spread was also low across these four studies (AUL: two, ALL: five, PLL: zero, and CD: three).

## Discussion

The duration of symptom relief is an important factor for patients receiving BoNTA injections; recent research has shown that the loss of symptom relief before the next injection has a severe impact on many aspects of quality of life (12, 13, 23). Across the four studies evaluated, aboBoNTA provided long-lasting relief from symptoms of spastic paresis or cervical dystonia in a substantial proportion of adult and pediatric patients as evidenced by the extended time to retreatment in these populations.

Since it is well-established that the duration of effect of BoNTA is positively correlated with the dose, which in turn correlates with the amount of active BoNTA delivered (9, 10), a possible explanation for the observed long time to retreatment in these studies conducted with Dysport could be the amount of active neurotoxin being administered to patients (22). The amount of active neurotoxin administered to patients varies between products, since each has a different approved dose—each approved dose being measured in potency units that differ and are non-interchangeable between products (10, 20, 24). Additionally, different *Clostridium botulinum* bacterial strains are used to produce the different products, and each has a different preparation and formulation (25).

**Figure 6:**
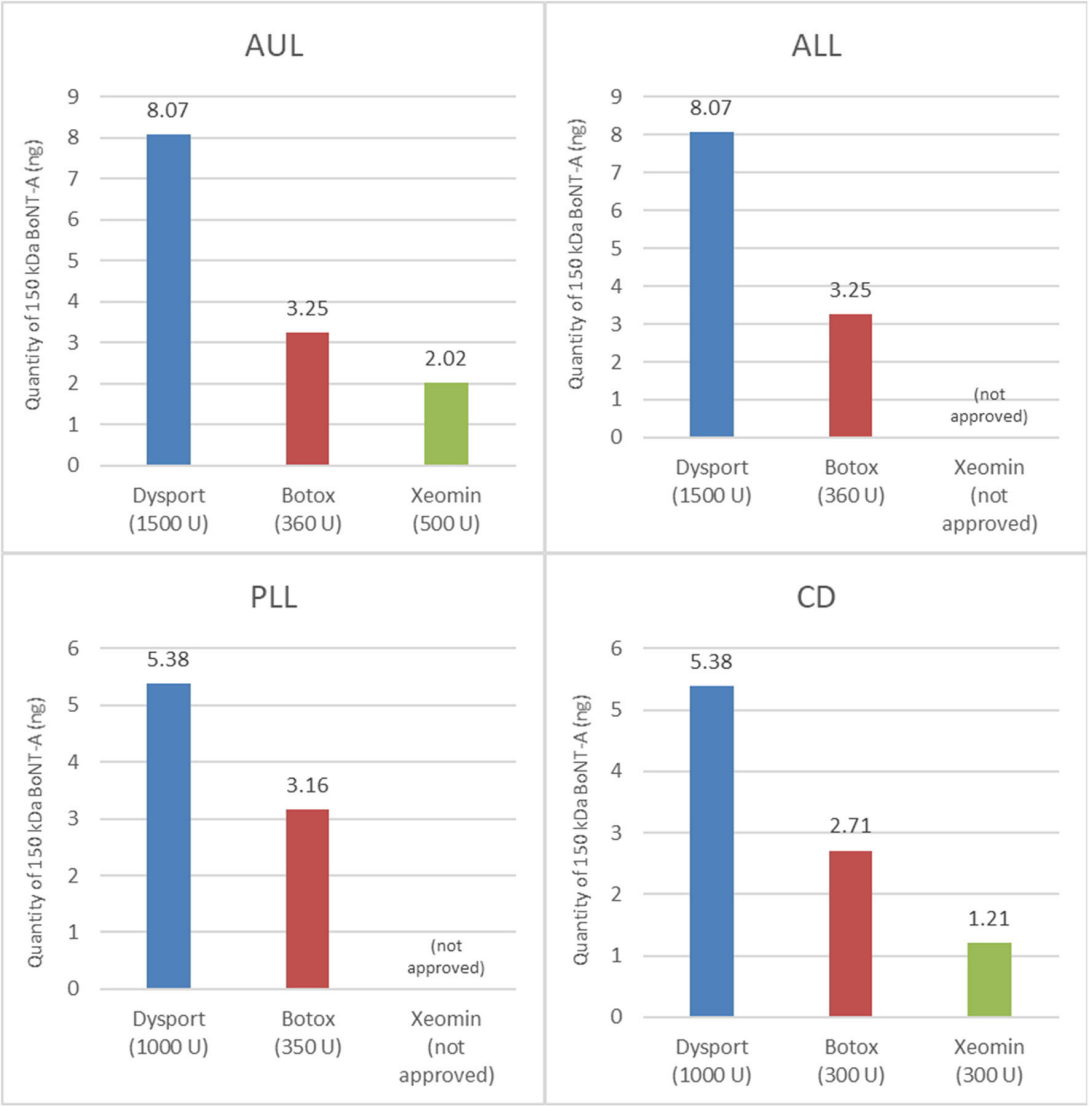
Quantity of active 150-kDa BoNTA in BoNTA products at their total recommended doses (22). The total recommended doses shown are for France.

Previous calculations have attempted to create conversion ratios between products (26–28), although no evidence-based ratio exists. Work by Frevert (29) has reported the relative amounts of toxin per product. This work was built on recently by Field et al. (22) who also provided a comparison of the quantity (ELISA assay) and the activity (EndoPep assay) of BoNTA in three commercial formulations (Botox®, Dysport®, and Xeomin®). In the research of Field *et al*., the ELISA assay was used to quantify the amount of 150-kDa BoNTA protein per unit (and per vial) of each product, and the EndoPep assay was used to quantify the catalytic light chain activity of BoNTA in the different products (specifically, its ability to cleave the SNAP-25 protein, which is how it exerts its effect on muscular activity). The results showed that there was no meaningful difference in the light chain activity between the three products and that the amount of 150-kDa BoNTA in each product represents the amount of active neurotoxin, while the mean 150-kDa BoNTA content per vial measured by ELISA was 2.69 ng/500 U vial of Dysport®, 0.90 ng/100 U vial of Botox®, and 0.40 ng/100 U vial of Xeomin®. When these values are adjusted for the approved doses in France (for example), it appears that greater amounts of active neurotoxin are injected with the approved doses of aboBoNTA than with the approved doses of the other products (Figure 6). We hypothesize that this observation might explain the long duration of action reported from clinical trials across multiple indications, which are presented in this paper.

The studies included here, together with others and a wealth of clinical consensus and practical experience, have demonstrated aboBoNTA to be well-tolerated, with equivalent safety and immunogenicity profiles compared with other BoNTA formulations. At the approved doses, there was no increase in adverse events with aboBoNTA compared with the adverse event profiles reported for other BoNT formulations (30–34). A mixed-treatment comparison evaluating four BoNTA treatments and one BoNTB treatment for cervical dystonia reported similar efficacy for all BoNT products at week 4, with all but a Chinese botulinum toxin serotype A (Prosigne®), which lacked effectiveness compared with placebo (35). Of the adverse events measured in this mixed-treatment comparison, neither dysphagia nor injection site pain was significantly greater in the treatment or in the placebo groups (35).

Taking the opposite perspective, we might ask what factors are associated with non-response—in particular, secondary non-response (i.e., patients experiencing non-response following a period of successful treatment with BoNTA). Secondary non-response may occur due to a number of reasons, including unrealistic expectations or use in an inappropriate indication (36, 37). It has also been hypothesized to be associated, at least in part, with the production of neutralizing antibodies; therefore, the propensity of a formulation and a regimen containing a foreign protein (such as botulinum toxin) to induce the production of neutralizing antibodies is a key component of the benefit analysis of BoNTA formulations (36). Antibody formation to BoNTA has only been noted rarely in patients receiving aboBoNTA (38), and a systematic analysis of BoNTA immunogenicity in clinical studies reported an overall rate of developing neutralizing antibodies to BoNTA of 2.1%, with differences being observed between some of the toxins [aboBoNTA 1.4%, incobotulinumtoxinA (incoBoNTA) 0.8–1.1%, old onabotulinumtoxinA (onaBoNTA) 7.2%, and new onaBoNTA 3.6%] (39). A recent systematic review found the rate of neutralizing antibodies in clinical studies of BoNTs for spastic paresis to be low (~1%), with no significant difference among formulations (37).

These findings could have important implications for dosing intervals as part of a personalized treatment strategy. Relevant to this, we note that preliminary results from the 3rd Upper Limb International Spasticity study suggest that our findings may also hold true in an observational, real-world clinical setting (40). In the first treatment cycle of that study, 410 patients treated with aboBoNTA had a mean time between first and second injections of 186.1 days—significantly longer than those treated with onaBoNTA (148.0 days, *n* = 158) and incoBoNTA (148.7 days, *n* = 72). Interestingly, relevant to the discussion above, the mean (SD) doses of BoNTA injected were 828.3 U (347.8 U), 235.3 U (123.0 U), and 271.5 U (139.0 U) for aboBoNTA, onaBoNTA, and incoBoNTA, respectively. Although, as mentioned above, it should be noted that the units are not interchangeable between products and cannot be compared directly, the longer duration between injections for aboBoNTA observed in this observational study may lend credence to the concept outlined above relating the amount of injected neurotoxin to the duration of effect. However, it should be noted that these preliminary results are based on an interim analysis of 80% of the patients; therefore, it will be important to ensure that these findings are corroborated by the full sample set from this study.

Limitations of this work could include the fact that it does not compare products head to head (in the absence of such trials having been run) and that the results are drawn from research clinical trials, which may not represent real-world dosing practices (albeit the real-world data cited above do appear to support our findings).

## Conclusions

In conclusion, aboBoNTA in open-label long-term trials provided a long duration of symptom relief in adults with upper or lower limb spasticity, children with lower limb spasticity, and adults with cervical dystonia. This may give patients sustained relief between injections and decrease the chance of patients experiencing a waning of effect before another treatment can be provided. This long duration of effect could lead to substantial improvements in the quality of life of patients and their caregiver/families and, potentially, reduced healthcare costs.

## Data Availability Statement

Where patient data can be anonymized, Ipsen will share all individual participant data that underlie the results reported in this article with qualified researchers who provide a valid research question. Study documents, such as the study protocol and clinical study report, are not always available. Proposals should be submitted to DataSharing@Ipsen.com and will be assessed by a scientific review board. Data are available beginning 6 months and ending 5 years after publication; after this time, only raw data may be available.

## Ethics Statement

The studies involving human participants were reviewed and approved by The work presents results from 4 studies—NCT01313299, NCT01249404, NCT01249417, and NCT00257660. All four received ethics board approval, the details of which are published elsewhere. The patients/participants provided their written informed consent to participate in this study.

## Author Contributions

All the authors have made substantial contributions to study conception/design or acquisition/analysis/interpretation of data, have helped to draft the manuscript or critically revise it for important intellectual content, and have given their final approval of its publication.

## Conflict of Interest

AE reports consultancy fees from Ipsen, Allergan, and Merz and research grants from Ipsen and Allergan. MD reports consultancy fees from Ipsen and Allergan and research grants from Ipsen. RH reports consulting fees from AbbVie, Acorda Therapeutics, Acadia, Adamas, AstraZeneca, ApoPharma, Cynapsus Therapeutics, Eli Lilly & Company, GE Healthcare, Impax Laboratories, Intec, Jazz Pharmaceuticals, Kyowa Kirin, Lundbeck LLC, The Lockwood Group, Medtronic, Michael J. Fox Foundation, Mitsubishi Tanabe Pharmaceuticals, Movement Disorder Society, National Institutes of Health (NIH), Neurocea LLC, Neurocrine Biosciences, Neuroderm, Neuropore Therapies, Pfizer, Sunovion, Sun Pharma, Teva Pharmaceutical Industries, and US WorldMeds. J-MG reports consultancy and research grants from Ipsen, Merz, and Allergan. AL is an employee of Ipsen. PP and KF were employees of Ipsen at the time of manuscript conception and development. The authors declare that this study received funding from Ipsen. The funder had the following involvement in the study: study sponsorship, study management.
