# Supplementary material for: Duration of Symptom Relief Between Injections for AbobotulinumtoxinA (Dysport®) in Spastic Paresis and Cervical Dystonia: Comparison of Evidence From Clinical Studies
Source: Front Neurol. 2020 Sep 25;11:576117. doi: 10.3389/fneur.2020.576117 (PMC7546809; doi:10.3389/fneur.2020.576117)
Supplement: Supplementary file 1 [file Data_Sheet_1.docx]

Duration of symptom relief between injections for AbobotulinumtoxinA (Dysport®) in spastic paresis and cervical dystonia

Alberto Esquenazi,^1^ Mauricio R. Delgado,^2^ Robert A. Hauser,^3^ Philippe Picaut,^4^ Keith Foster,^5^ Andreas Lysandropoulos,^6^ Jean-Michel Gracies^7^

^1^ Department of Physical Medicine and Rehabilitation, MossRehab Gait and Motion Analysis Laboratory, Elkins Park, PA, USA.

^2^ Neurology and Neurotherapeutics Department, University of Texas Southwestern Medical Center and Scottish Rite Hospital for Children, Dallas, TX, USA.

^3^ University of South Florida Parkinson's Disease and Movement Disorders Center of Excellence, Tampa, FL, USA.

^4^ Ipsen Pharma, Cambridge, MA, USA.

^5^ Ipsen Bioinnovation, Abingdon, UK.

^6^ Ipsen, Boulogne-Billancourt, France.

^7^ EA 7377, Université Paris-Est Créteil, Service de Rééducation Neurolocomotrice, Albert Chenevier-Henri Mondor Hospital, APHP, 94000 Créteil, France.

*** Correspondence:**Alberto Esquenazi
[AESQUENA@einstein.edu](mailto:AESQUENA@einstein.edu)

# Supplementary Material

We provide herein further details on the methodologies of the four studies reported in this article. Please note that full details can be obtained in the original publications (Gracies et al 2015; 2017a,b; Delgado et al 2016; Truong et al 2010) but we collate this together here for the convenience of the reader.

Data were taken from four large, phase 3, international, randomised, placebo-controlled studies with open-label extension phases involving aboBoNTA in adults with cervical dystonia (Truong et al 2010), adults with upper or lower limb spasticity following a stroke or traumatic brain injury (Gracies et al 2015, 2017a,b), and children with lower limb spasticity as a result of cerebral palsy (Delgado et al 2016).

**Double-blind and open-label studies in adult patients with upper limb spasticity**

This was a Phase 3, international, multicentre, double-blind, randomised, single-cycle study of aboBoNT-A in adults with upper limb spasticity, followed by a long-term open-label, multiple-cycle extension study (NCT01313299; see **Figure 1**). Open-label study patients received up to four additional treatment cycles over a maximum of 1 year (number of injections varied depending on patient needs).

Key inclusion criteria for the double-blind study were: hemiparetic adults aged 18–80 years, ≥6 months post-stroke or traumatic brain injury (TBI); Modified Ashworth Scale (MAS) score ≥2 (toxin-naïve patients) or 3 (toxin-non-naïve patients) in the primary target muscle group (PTMG); Disability Assessment Scale score ≥2 in principle target of treatment; spasticity angle ≥10° in PTMG; overall Modified Frenchay Scale (evaluating performance of ten upper limb tasks) score of between 1 and 8 (rated on a 10-point scale).

Key inclusion criteria for the open-label extension were: rollover patients who completed the double-blind study with no major protocol deviations or ongoing TEAEs; and newly recruited patients (i.e., patients not participating in the double-blind study) met the same criteria defined for the double-blind study.

In the double-blind phase, patients were randomised to receive a single treatment cycle of placebo, 500 U or 1000 U aboBoNTA into the PTMG (elbow, wrist or extrinsic finger flexors) and at least two other upper limb muscles, as selected by the investigator (see Figure 1). In the open-label extension phase, at treatment cycle 1, patients were administered 1000 U aboBoNTA; patients who experienced adverse events in the double-blind study received 500 U or 1000 U aboBoNTA at the investigator’s discretion. At subsequent treatment cycles, patients could receive 500 U aboBoNTA into the shoulder muscles (total body dose ≤1500 U), and from treatment cycle 3, concomitant 500 U injection of aboBoNTA into lower limbs was permitted (total body dose ≤1500 U).

At each treatment cycle, retreatment was per the investigator’s clinical judgement and was possible at Weeks 12, 16, 20 and 24.

**Double-blind and open-label studies in adult patients with lower limb spasticity**

This was a Phase 3, international, multicentre, prospective, double-blind, randomised, single-cycle study of aboBoNTA in adults with lower limb spasticity (NCT01249404), followed by a long-term, open-label, multiple-cycle extension study (NCT01251367; see **Figure 1**). Open-label study patients received up to four additional treatment cycles over a maximum of 1 year (number of injections varied depending on patient needs).

Key inclusion criteria in the double-blind study were: adults with spastic hemiparesis causing gait deficiency; 18–80 years of age; ≥6 months post-stroke or TBI; MAS score ≥2 or 3 in the gastrocnemius–soleus complex (GSC) for toxin-naive and non-toxin-naïve patients, respectively; spasticity angle ≥5° in the GSC (Tardieu scale); comfortable barefoot walking speed between 0.1 and 0.8 m/s at baseline.

Key inclusion criteria for the open-label extension were: patients who completed the double-blind study with no major protocol deviations or ongoing treatment-emergent adverse events (TEAEs).

In the double-blind phase, patients were randomised to receive a single treatment cycle of placebo, 1000 U or 1500 U aboBoNTA into both soleus and gastrocnemius muscles and at least one other lower-limb muscle selected by the investigator (**Figure 1**). In the open-label extension, at treatment cycle 1, all patients received 1500 U aboBoNTA. At subsequent treatment cycles, patients were administered 1000 U or 1500 U aboBoNTA at the investigator’s discretion. From treatment cycle 3, concomitant 500 U injection of aboBoNTA into upper limb was allowed (total body dose remained ≤1500 U).

At each treatment cycle, retreatment was per investigator’s clinical judgement and was possible at Weeks 12, 16, 20 and 24. At each study visit from Week 12 onwards of each treatment cycle, the following conditions were assessed by the investigator to determine the timing of the next treatment cycle:

- If the patient had not demonstrated a decrease from baseline of at least one grade in the MAS score in the GSC (knee extended), and had no improvement from baseline on the Physician’s Global Assessment (PGA; i.e. a score ≤0) and if, based on the investigator's judgement, there was no unacceptable safety risk to the patient to receive the next treatment cycle, the patient was injected on the same study day (i.e. Week 12).
- If the patient had a decrease from baseline of at least one grade in the MAS score in the GSC and/or had demonstrated improvement from baseline on the PGA (i.e. a score ≥+1), the investigator decided, based on other efficacy and safety criteria, whether the subject needed to be injected on the same day (i.e. Week 12) or whether injection was postponed to the next visit.
- If the subject experienced any unacceptable risk, the subject was discontinued.

Thus, if a patient was not re-injected, the MAS/PGA was at least stable or improved. Hence, the time to reinjection reflects the duration of effect of aboBoNTA.

**Double-blind and open-label studies in paediatric patients with lower limb spasticity**

This was a Phase 3, international, multicentre, double-blind, prospective, randomised, placebo-controlled, single-dose study (NCT01249417; see Figure 1). Key inclusion criteria were: ambulatory children aged 2–17 years with a diagnosis of spasticity due to cerebral palsy and equinus foot positioning during the stance phase of the gait; Gross Motor Function Classification System Level of I–III; MAS ≥2 and a spasticity grade (Y) of 2–4 on the Tardieu scale (with a spasticity angle [X] of 10° or more) at the ankle joint; no fixed ankle flexion myocontractures, previous surgery, or alcohol/phenol injections; no serial casting in the past 12 weeks; and BoNT naïve or a minimum of 6 months’ washout from the last BoNT injection for any condition.

All patients who successfully completed the double-blind study and who continued to meet eligibility criteria could enter the open-label phase of the study (NCT01251380; see **Figure 1**). During the double-blind study, patients received injections of placebo, 10 U/kg/leg aboBoNTA (i.e. 20 U/kg for bilateral injections) or 15 U/kg/leg aboBoNTA (i.e. 30 U/kg for bilateral injections) into the gastrocnemius and soleus muscles. The maximum total dose was 30 U/kg or 1000 U, whichever the lower value.

Following treatment administration, patients attended follow-up visits at Weeks 4 and 12. Additional visits to evaluate eligibility criteria for retreatment were permitted at Week 16 (for patients who in the clinical judgment of the investigator did not require retreatment at Week 12), at Week 22 (patients who did not require retreatment at Week 16) and at Week 28 (patients who did not require retreatment at Week 22).

If assessed as eligible for retreatment, patients were then included in the open-label study. Eligibility for retreatment was defined as either:

- if the patient has not demonstrated a decrease from baseline of ≥1 grade in the MAS score in the GSC at the ankle joint and has no improvement in PGA score (i.e. score ≤0), and if based on the investigator’s judgement there is no unacceptable safety risk; or
- patient has demonstrated a decrease from baseline of ≥1 grade in the MAS score in the GSC at the ankle joint and/or has demonstrated an improvement on the PGA (i.e. a score ≥+1), the Investigator will decide based on the other efficacy and safety assessments whether the patient needs to be injected on the same day or whether the injection is postponed to the next scheduled visit (i.e. Week 16, 22, 28 or later).

The minimum retreatment interval was 12 weeks.

**Double-blind and open-label studies in patients with cervical dystonia**

This was a Phase 3, multicentre, double-blind, prospective, randomised, placebo-controlled, single-dose study with an open-label extension phase (NCT00257660 and NCT00288509; see **Figure 1**).

Key inclusion criteria for the double-blind study were: adults aged ≥18 years; history of cervical dystonia within 18 months of onset; toxin-naïve or at least 16 weeks since last BoNT injection; Toronto Western Spasmodic Torticollis Rating Scale (TWSTRS) total score ≥30; TWSTRS Severity sub-scale score ≥15; TWSTRS Disability sub-scale score ≥3; TWSTRS Pain sub-scale score ≥1. Patients who completed the double-blind phase were eligible for the open-label phase.

During the double-blind treatment, patients received a total dose of 500 U into two to four of the clinically indicated neck muscles in a single dosing session, with or without electromyogram guidance (according to the investigator’s normal practice). The number of injection sites and dose were determined by the investigator. In the first dose of the open-label phase, patients received 500 U but doses in subsequent treatment cycles could be titrated to the individual patient (250–1000 U).

**Endpoints of this analysis**

Across all of the studies reported here, the endpoints were time to retreatment (the visit at which patients were reinjected), and safety (adverse events reported and immunogenicity).

Descriptive statistical analyses are presented throughout.
